# Attention-Deficit/Hyperactivity Disorder, Imposter Phenomenon, and Identity Distress: The Mediating Indirect Effects of Self-Esteem, Social Camouflaging, and Social Media Connections

**DOI:** 10.3390/bs16020213

**Published:** 2026-01-31

**Authors:** Julie M. Hall, Aubrianna L. Stuckey, Steven L. Berman

**Affiliations:** Department of Psychology, University of Central Florida, Sanford, FL 32773, USA; aubrianna.stuckey@ucf.edu

**Keywords:** Attention-Deficit/Hyperactivity Disorder, imposter phenomenon, social camouflaging, self-esteem, identity distress

## Abstract

The previous literature has explored the various relationships among Attention-Deficit/Hyperactivity Disorder (ADHD), identity distress, imposter phenomenon (IP), self-esteem, masking, and social media, but to our knowledge no studies have looked at all the variables together within in a single model. This study aimed to test the fit of a structural equation model (SEM) exploring the direct relationships between ADHD symptom severity, IP, identity distress and the mediating indirect effects of self-esteem, social camouflaging, and social media connections. Specifically, we tested if self-esteem, masking, and social media connections mediate the pathways between ADHD and IP and ADHD and identity distress. College students (*N* = 500, women 61.6%, men 34%) completed an anonymous online survey battery. Those whose self-report symptom scores suggested that they might meet the DSM-5-TR criteria for ADHD had higher levels of IP, integration of social media use for communication, and identity distress and lower levels of self-esteem compared to students whose scores suggested that they probably would not meet the criteria for ADHD. A significant path was found from ADHD symptom severity to IP and to identity distress mediated through self-esteem, masking, and social media connections (emotional connection to social media and integration into life). This study is among the first to explore these relationships, in hopes of further informing clinicians’ planning prevention and intervention strategies for those who are struggling with ADHD and identity issues. Further results and their implications are discussed.

## 1. Introduction

Platforms such as TikTok and Instagram have influenced how individuals understand psychological conditions, including Attention-Deficit/Hyperactivity Disorder (ADHD). Social media has indicated that imposter phenomenon (IP) is strongly related to ADHD, while previous research has shown that IP is closely related to biological sex (mainly females) and self-esteem (Clance, 1985; Luyckx et al., 2013). While social media platforms provide validation, community, and education for those identifying with ADHD traits (Eagle & Ringland, 2023), they are also known to facilitate comparison, misinformation, and unrealistic standards of success (Yeung et al., 2022; Saiphoo et al., 2020). This paradoxical role of social media—as both a source of belonging and self-doubt—may amplify experiences of imposterism among individuals with ADHD symptomatology. Moreover, those with ADHD may engage in *social masking* or *camouflaging* behaviors—in efforts to appear more neurotypical in social contexts—which are associated with identity distress and emotional exhaustion (Hull et al., 2019; van der Putten et al., 2024).

Individuals with ADHD frequently report lower self-esteem and higher identity distress compared to neurotypical peers (Berman, 2020; Molavi et al., 2020). According to Berman (2020), identity distress can be characterized by negative feelings related to an individual’s inability or difficulty resolving identity issues (e.g., vocational choices, long-term goals, personal values). Similarly, IP is strongly tied to low self-esteem and instability in self-concept (Schubert & Bowker, 2019). Despite these intersections, no published research has systematically investigated whether ADHD symptom severity predicts imposter feelings or how self-esteem, masking, and social media use might explain this relationship.

The current study aims to address this gap by exploring the relationships among ADHD symptom severity, imposter phenomenon, self-esteem, social camouflaging, identity distress, and social media integration in a college student sample. This study contributes novel empirical insight into how internal experiences of ADHD intersect with social and digital contexts to shape self-perception and identity during a key developmental phase of life.

### 1.1. The Current Study

It was long believed that ADHD was a childhood neurodevelopmental disorder that individuals eventually grew out of, but it is now widely recognized as a disorder that persists throughout adulthood (Swanson et al., 2016). While there has not been research conducted to explore the possible link between ADHD and IP, there are many overlapping factors of ADHD that could contribute to the risk of IP. Clance (1985) first noted IP in clinical therapeutic sessions with high-achieving women and chose to refer to the symptoms as IP instead of imposter syndrome due to not wanting another syndrome to be associated with women. IP is a failure to internalize personal success (Clance & Imes, 1978). IP occurs when a person feels like a fraud or as if they are incompetent, though the evidence would show that they are generally successful by external standards (Chrisman et al., 1995).

Many different factors have been found in relation to IP, including socioeconomic status and family, ethnicity, and personality (Bravata et al., 2020). Similarly, factors such as social camouflaging and low self-esteem, which are common features of ADHD, are also attributed to higher levels of IP. Symptoms have been found to be more prominent in transitional situations, such as starting college or beginning a new job (Topping & Kimmel, 1985). IP has also been found to be related to perfectionism and low self-esteem (Clance, 1985; Schubert & Bowker, 2019). Rakestraw (2017) notes that the most significant difference between self-esteem and IP is that low self-esteem tends to be pervasive in multiple areas of a person’s life. In contrast, IP is prevalent in specific areas of one’s life, such as in work or school. A systematic review of IP carried out by Bravata et al. (2020) found that there were conflicting data on gender and age effects but that IP tends to be comorbid with depression, anxiety, low self-esteem, negative internal dialogue, somatic symptoms, and social dysfunction. Additionally, the researchers noticed significant differences between peer-reviewed articles and non-scholarly literature, such that academic articles use the term “imposter phenomenon”, whereas non-scholarly literature prefers the term “imposter syndrome”. Non-scholarly literature also gives advice on how to manage symptoms. In contrast, academic literature does not have a specific treatment for IP and instead focuses on treating the comorbid conditions (Bravata et al., 2020).

Koyuncu et al. (2018) recognize that there are a few maladaptive social behaviors displayed by individuals with ADHD, such as errors, blunders, and forgetfulness, which lead to criticism from peers and adults, which in turn leads ADHD individuals to fear criticism and failure. One such way individuals with ADHD are more likely to attempt to circumvent these criticisms is by utilizing social camouflaging. Social camouflaging, more colloquially known as masking, is defined by the neurodivergent community as attempting to act or present as neurotypical in social situations (Lei et al., 2019). Neurodivergent is a common term used to describe those who fall outside the realm of normal neurocognitive and neurodevelopmental societal norms, such as individuals with ADHD (Shah et al., 2022). Social camouflaging in the neurodiverse community consists in suppressing and attempting to control “socially undesirable behaviors”. It is generally characterized by effortful and compensatory behaviors that can minimize a person’s true self (Lei et al., 2019), yet it can also be utilized unknowingly by neurodivergent people as a way to blend in better with a neurotypical world (Hull et al., 2017). Those with ADHD have been known to be stigmatized as socially undesirable, which is possibly why they also “mask” (Ai et al., 2024).

The negative feedback due to these social blunders can also contribute to low self-esteem and a fear of letting others truly know them (Koyuncu et al., 2018). These negative social experiences and fear of letting others see their true selves could, in turn, lead to feelings of IP despite any achievements earned. Self-esteem was defined by Rosenberg (1979) as the general and evaluative regard in which people hold themselves, either in a positive or negative way or from low to high. Self-esteem is found to be a subjective personal evaluation of one’s own worth and tends to be relatively stable over a lifetime (Donnellan et al., 2012; Robins & Trzesniewski, 2005).

Harpin et al. (2016) found that those with ADHD tend to have lower self-esteem than their non-ADHD counterparts, and low self-esteem has been shown to contribute to IP (Schubert & Bowker, 2019). Molavi et al. (2020) found that those with cognitive impairments along with ADHD had lower levels of self-esteem, and those with predominantly inattentive-type or combined-type ADHD were more likely to struggle with cognitive impairments and low self-esteem than those with predominantly hyperactive/impulsive type.

Brown and Mankowski (1993) reported that deficits in self-esteem can lead to problems with identity, which can lead to cognitive, motivational, emotional, and interpersonal issues. According to Berman (2020), numerous studies have shown that 8% to 12% of adolescents and emerging adults have debilitating identity problems and that there is a correlation among college students between symptom severity of adjustment problems and identity distress. While there is a natural developmental stage in life when people must make decisions about their lives, it is when the severity of these uncertainties increases to the point of impairment that it can be considered an identity crisis. For example, Luyckx et al. (2013) found that self-esteem and identity have a positive correlation and that the stronger self-identity one possesses, the higher self-esteem one tends to hold; likewise, they found that lower self-esteem correlated with a weaker sense of identity. Self-esteem could be a common factor between ADHD and identity distress. While no studies to date have specifically evaluated the relationship between ADHD, identity distress, and self-esteem, it has been established that people with ADHD tend to develop lower levels of self-esteem during childhood and adolescence that continues into adulthood (Molavi et al., 2020). Due to the fact that identity issues are most salient during late adolescence to early adulthood (Erikson, 1968), studies that aim to focus on identity issues most frequently focus on that demographic age group.

Recent studies have examined the effects of social media on people who have ADHD. Eagle and Ringland (2023) found that social media is a place where people with undiagnosed ADHD have begun to share experiences or challenges associated with ADHD and have found validation and acceptance in online communities. Looking specifically at Twitter, TikTok, and Instagram, they suggested some pros and cons of these platforms. Some of the positives of these platforms included increasing patient knowledge of the disorder and encouraging self-advocacy, providing psychological support, and creating a community. Sharing of misinformation regarding mental health, knowingly or unknowingly, can be harmful, leading to distorted or false beliefs (Flynn et al., 2017). On TikTok specifically, Yeung et al. (2022) systematically reviewed the top 100 ADHD videos and found that 52% of the information shared about ADHD was inaccurate, and the misleading videos were more likely to be uploaded by non-healthcare providers. They also found that 27% of the top 100 videos were personal experiences, and only 21% were seen as useful. Some of the common misinformation seen on social media attributes normal conditions of the human experience, such as having trouble focusing or being able to blink in count to a song, as signs of the disorder (Eagle & Ringland, 2023).

Smith and Anderson (2018) found that 94% of college-aged adults reported using social media. Dekkers and van Hoorn (2022) found that adolescents who displayed symptoms of ADHD were more likely to display a higher intensity (or more frequent) and problematic use of social media. Settanni et al. (2018) found that among adolescents, higher levels of ADHD symptoms were predictive of higher levels of addictive Facebook use. Studies have also shown that social media can be addictive and that social media use is prevalent when driving, especially in people who exhibit ADHD symptoms (Turel & Bechara, 2016). Hartnett and Cummings (2023) suggest that social media platforms have the potential to influence users, warning that this might create social contagion and induce illness behavior. A meta-analysis exploring social networking site (SNS) use and self-esteem found that there was a small, negative, yet significant relationship between social media use and self-esteem (Saiphoo et al., 2020). Higher levels of SNS use, specifically more addictive type use, were related to lower levels of self-esteem. Myers (2021) found conflicting views on social media and IP, where some viewed social media as a way to compare themselves to others, and others used it as a way to share their accomplishments.

### 1.2. Rationale

The previous literature has explored the various relationships among ADHD, identity distress, imposter phenomenon, self-esteem, masking, and social media, but to our knowledge no studies have looked at all the variables together within a single model. Therefore, the purpose of this study was to explore the relationships among these variables.

Bravata et al. (2020) found a significant relationship between IP and job performance, job satisfaction, and burnout. El-Ashry et al. (2024) found that IP was strongly positively correlated with depression, stress, and anxiety, though specifically in nursing students. Orth and Robins (2014) found that self-esteem is a relatively stable trait throughout a life span, starting at adolescence through adulthood, generally peaking between 50 and 60 years of age. The authors also found that self-esteem predicts future outcomes in different domains of life, including work, health, and relationships. Higher levels of self-esteem are related to success and well-being in these areas of life (Orth & Robins, 2014). Slomkowski et al. (1995) were among the first researchers to directly suggest that social functioning and self-esteem may affect the relationship between ADHD, specifically hyperactivity, and future outcomes, such as educational achievements and occupational ranks. Also, those with ADHD have been known to be stigmatized as socially undesirable, which is possibly why they also “mask” (Ai et al., 2024).

Identity problems or identity distress encompasses negative feelings such as anxiety and depression around the inability to resolve certain identity issues (Berman, 2020). While there is a natural developmental stage in life when people must make decisions about their lives, it is when the severity of these uncertainties increases to the point of impairment that it can be considered an identity crisis. Realms of identity that can cause anxiety and distress include long-term goals, career choice, sexual orientation, religious affiliation, morals and values, friendships, and group identities (American Psychiatric Association, 1980). Due to the outcomes associated with the different variables, the current study aims to advance understanding of the internal processes associated with ADHD and these variables in emerging adulthood. Clarifying these relationships could have important implications for future assessments, psychoeducation, and interventions for individuals with ADHD.

**Hypothesis** **1.** *It was hypothesized that ADHD symptom severity would be a core predictor of both IP and identity distress.*

**Hypothesis** **2.** *The relationship between ADHD symptom severity and outcome variables (IP and identity distress) would be mediated by self-esteem, masking, and social media connections.*

## 2. Materials and Methods

### 2.1. Participants

Participants were recruited through convenience sampling of college students who were enrolled in a large southeastern metropolitan university (*N* = 500). Students found the survey through SONA, the university’s online recruitment platform. Students ranged between 18 years and 66 years of age (*M* = 20.05, *SD* = 4.49). The sample of students consisted of women (61.6%), men (34%), non-binary (1.6%), transgender (1.2%), and “other” or chose not to disclose (1.6%). More than half of the sample was non-Hispanic White (52.4%), with the remainder consisting of Hispanic or Latino/a (25.2%), Asian or Pacific Islander (9.4%), Black, non-Hispanic (6.2%), Native American or Alaskan American (0.4%), and Multi-Ethnic or “other” (6.4%). Grade level distribution was 43.6% Freshmen, 26.8% Sophomores, 16.2% Juniors, 11.8% Seniors, 0.4% non-degree seeking, 0.2% Graduate students, and 1% identified as “other”. Throughout the survey battery, attentional checks were distributed to ensure response validity and gauge survey burnout. Participants had to be over the age of 18 to participate.

### 2.2. Measures

#### 2.2.1. Demographics

A demographic questionnaire inquired about gender identity, age, ethnicity, grade level, and prior ADHD diagnosis.

#### 2.2.2. Social Media Use Integration Scale (Jenkins-Guarnieri et al., 2012)

This 10-question measure was originally designed to determine how integrated Facebook use was in people’s lives. The wording was changed in this study by substituting the word Facebook with “Social Media” to be more encompassing of different social media platforms. This measure has two factors: social integration and emotional connection, and integration into social routines. It is rated on a five-point scale of 1 = Strongly Disagree, 2 = Disagree, 3 = Neutral, 4 = Agree, and 5 = Strongly Agree. Responses on the items were averaged for a score. In this study, this scale had a Cronbach’s alpha of 0.87 for the total scale, 0.79 for emotional connection, and 0.83 for social integration.

#### 2.2.3. Adult ADHD Self-Report Scale (ASRS v1.1; Adler et al., 2006)

This symptom checklist consists of eighteen questions based on DSM-IV-TR criteria for ADHD diagnosis. It also has been found to be consistent with DSM-5-TR criteria (Kessler et al., 2005). Although a self-report measure alone, without a diagnostic interview, would be inappropriate to diagnose with certainty, this was carried out for research purposes to establish groups likely and unlikely to meet the criteria. Four questions were added to the scale to better meet DSM-5-TR symptom criteria and included the following: “How often do you feel as though these symptoms affect you in multiple settings (e.g., at home, school, work, etc.)”, “How often do you feel as though these symptoms interfere with, or reduce your quality of social, academic, or occupational functioning”, “Have you ever been diagnosed with a disorder that was/is not ADHD that explains these symptoms?”, and “If you answered anywhere from “sometimes” to “very often” for any of the above symptoms, about how old were you when you first started to experience or notice these symptoms?” The measure has a five-point scale (0 = never, 1 = rarely, 2 = sometimes, 3 = often, 4 = very often) to indicate the frequency of symptoms experienced. In this study, a Cronbach’s alpha of 0.89 was found for the total score, 0.84 for predominantly inattentive, and 0.82 for predominantly hyperactive/impulsive.

#### 2.2.4. Clance Imposter Phenomenon Scale—Short Form (Chrisman et al., 1995)

This seven-question survey measures whether individuals have characteristics of imposter phenomenon. It includes questions such as “When people praise me for something I’ve accomplished, I’m afraid I won’t be able to live up to their expectations of me in the future.” It is rated as 1 = Very Rarely, 2 = Sometimes, 3 = Often, and 4 = Almost Always. Responses on the items were averaged to determine a total score. In this study, it was found to have an internal consistency reliability of 0.81.

#### 2.2.5. The Rosenberg Self-Esteem Questionnaire (Rosenberg, 1965)

The self-esteem questionnaire is a 10-item scale that measures people’s positive and negative feelings about themselves. All items are rated on a five-point Likert scale (1 = Strongly Disagree, 2 = Disagree, 3 = Neutral, 4 = Agree, 5 = Strongly Agree) and include questions such as “I feel that I have a number of good qualities.” In this study, the Cronbach’s alpha was found to be 0.90.

#### 2.2.6. Identity Distress Scale (Berman et al., 2004)

This scale assesses an individual’s level of distress and discomfort in the process of identity development. It is a seven-item survey that asks participants the following: “to what degree have you been upset, distressed, or worried over any of the following issues in your life: long-term goal, career choice, friendships, sexual orientation or behavior, religion, values or beliefs, and group loyalties.” Questions are answered on a five-point scale (1 = None at all, 2 = Mildly, 3 = Moderately, 4 = Severely, 5 = Very Severely) with regard to the degree of distress. Responses to the items were averaged for a total score. In this study, the measure had a Cronbach’s alpha of 0.77.

#### 2.2.7. Camouflaging Autistic Traits Questionnaire (Hull et al., 2019)

This 25-question self-report was originally developed to measure social camouflaging behavior in individual adults with ASD. There is currently no measure of social camouflaging that has been created for a more generalized population or specifically for those with ADHD (van der Putten et al., 2024). This measure is scored on a seven-point Likert scale (1 = Strongly Disagree, 2 = Disagree, 3 = Somewhat Disagree, 4 = Neither Agree nor Disagree, 5 = Somewhat Agree, 6 = Agree, 7 = Strongly Agree). Questions such as “When I am interacting with someone, I deliberately copy their body language or facial expressions” is an example of compensation, while “I monitor my body language or facial expressions so that I appear relaxed” demonstrated “masking” and “In social situations, I feel like I am pretending to be ‘normal’” reflect assimilation. Responses on the items were averaged to create a total score. In this study, the internal consistency was found to be 0.90 for the total scale, as well as 0.85 for compensation, 0.74 for masking, and 0.86 for assimilation factors.

### 2.3. Procedure

The proposal was first submitted to the authors’ institutional review board for approval. After receiving approval, this study was set up through SONA, a participant recruitment system. Participants earned course credit for their participation. The surveys were conducted online and anonymously. If students did not wish to participate in this study, they could select other studies or complete an alternate assignment with similar time and effort required for equivalent credit.

## 3. Results

### 3.1. Analyses

This study utilized SPSS 29 (IBM Corp., 2023) software to run all analyses. Pearson product correlation was utilized to determine if any study variables were associated with age. Two independent sample *t*-tests were conducted to examine any preliminary differences in outcome variables. The first *t*-test examined differences between men and women. The second *t*-test examined differences between participants who met ADHD criteria and those who did not meet criteria.

To determine if there were any ethnic/racial differences or differences in year in school present among the variables, One-Way Analysis of Variance (ANOVA) tests were conducted. The main analysis was run as a structural equation model (SEM) using AMOS 29.0 software.

### 3.2. Preliminary and Descriptive Results

A series of analyses were conducted to determine if there were any differences in the study variables by demographic categories. The dependent variables that correlated significantly with age were identity distress (*r* = −0.10, *p* = 0.027) and social media integration (*r* = −0.25, *p* < 0.001). The bivariate correlation matrix also highlights patterns of significance between other variables within this study, which can be found in Table 1.

Independent *t*-tests revealed significant differences between all study variables except for self-esteem, as seen in Table 2. While the initial *t*-test showed significant differences between genders, the final SEM model did not show significant differences.

The only ethnic differences were found for self-esteem (*F*_(4, 487)_ = 2.84, *p* = 0.024). A Tukey HSD post hoc analysis revealed that White non-Hispanics scored significantly higher in self-esteem than Asian/Pacific Islander (*p* = 0.03), and Hispanic/Latino/a also scored significantly higher than Asian/Pacific Islander (*p* = 0.013). There were no other significant differences between groups. Additionally, no differences were found between participants’ year in school on any of the study variables.

Participants were also questioned on whether or not they had a prior diagnosis of ADHD by a medical or psychological professional; 87% responded no, and 13% responded yes. The Adult ADHD Self-Report Scale (Adler et al., 2006), along with four additional questions, was also used to determine if students might meet criteria for DSM-5 ADHD diagnosis, in which 91.4% did not meet criteria, and 8.6% endorsed symptoms that suggested that they might meet criteria. These students were also broken into subtypes in which 7.6% reported symptoms that suggested that they would meet criteria for predominantly inattentive type, and 5.4% would meet criteria for predominantly hyperactive/impulsive type. These statistics compare to data from the National Center for Health Statistics Rapid Surveys System collected during October–November 2023, which showed that approximately 6% of US adults had a current diagnosis of ADHD (Staley et al., 2024).

Those whose responses suggest that they might meet criteria for ADHD scored significantly higher in imposter phenomenon, identity distress, masking, social media use integration, and significantly lower in self-esteem (See Table 3).

### 3.3. Main Analyses

**Figure 1:**
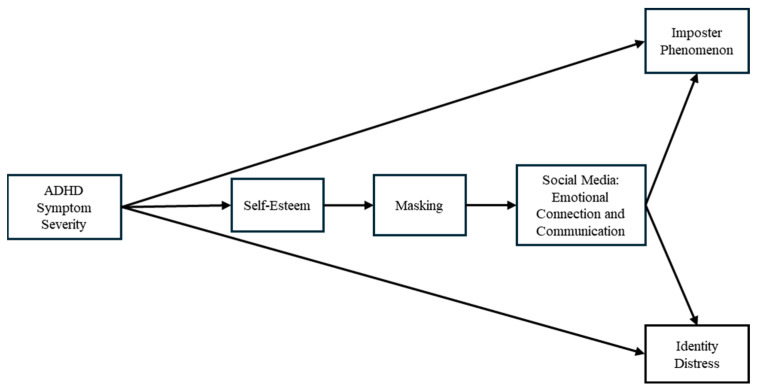
Proposed Structural Equation Model for Hypothesis.

An SEM was conducted to test the relationship paths between ADHD symptom severity and two outcome variables (imposter phenomenon, identity distress), while accounting for the potential mediating effects of self-esteem, masking, and social media connections (Figure 1).

**Figure 2:**
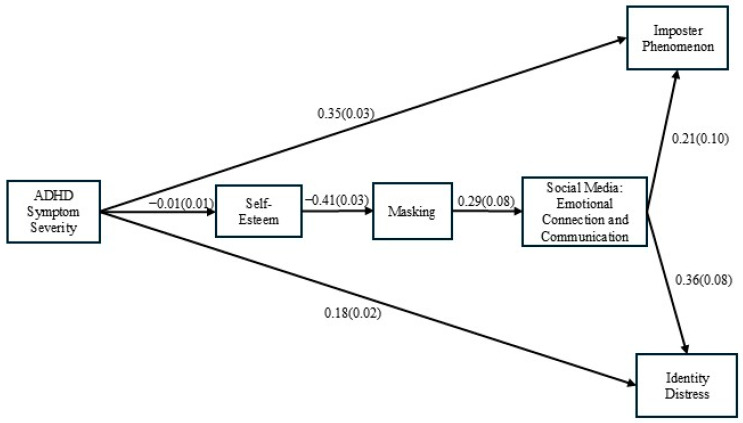
Structural Equation Model for Hypothesis Results.

The proposed model was an acceptable fitting model for the data set with a chi-squared value of *χ*^2^_(85)_ = 506.07, *p* < 0.001, with a CMIN/DF ratio of 5.94, root-mean-square error of approximation (RMSEA) = 0.10 (90% CI [0.09, 0.11]), CFI = 0.88, and TLI = 0.82. Inspection of the residuals and modification indices revealed no ill-fit in the model. Figure 2 is a model highlighting the significant paths and includes parameter estimates for the structural coefficients in the model. Estimate coefficients appear on each path, with standard estimates in parentheses. Standardized coefficients for all paths in the model and significance levels can be found in Table 4.

## 4. Discussion

Most research surrounding ADHD has focused more on functioning and behavioral or observable consequences of the disorder, especially among younger males. This project aimed to expand on often overlooked concepts such as the internal consequences of ADHD, like self-esteem, identity distress, and imposter phenomenon, while also exploring the role that social media integration use in one’s life might play in these relationships.

In recent years, there has been a trend on social media and non-scholarly websites to claim that many with ADHD are at higher risk of IP. Despite these claims, there have been no empirical studies that explore this relationship, to our knowledge. The current findings provide support to these assertions by demonstrating a strong relationship between ADHD symptom severity and IP, while highlighting the fact that there is no direct causation.

The results of this study were consistent with the previous literature in that the present findings indicated that stronger social media use and connection was associated with higher levels of both identity distress and IP. These results suggest that social media may act as a reinforcer for feelings of imposterism and perpetuate identity-related concerns, especially for those who have higher ADHD symptom severity, struggle with low self-esteem, and have higher masking tendencies.

Ultimately, the SEM supported a dynamic, multivariate framework in which ADHD symptom severity exerted both a direct and indirect effect on identity distress and IP. Multiple predictor variables were specified as exogenous and were allowed to covary, which reflected the interconnected nature of these variables. These findings suggest that identity distress and IP are not driven by a single factor but rather are driven by multiple processes including self-esteem, masking, and social media use. A significant pathway from ADHD symptom severity to both identity distress and IP through these mediators was identified, therefore fully supporting this study’s hypothesis.

There are many reasons why a person with ADHD might be more likely to experience IP and identity distress including self-esteem (Koyuncu et al., 2018), masking tendencies (Ai et al., 2024), and even the use of social media (Saiphoo et al., 2020). Koyuncu et al. (2018) recognized that there are many external social factors that could lead to a person with ADHD having lower self-esteem. Schubert & Bowker (2019) found that low self-esteem can contribute to IP. Consistent with the literature, the present model demonstrated that higher ADHD symptom severity was associated with lower self-esteem, which then contributed to increased social camouflaging. Social rejection and negative feedback from others can lead people with ADHD to mask (Ai et al., 2024). Lebowitz (2016) found that even the perception of having ADHD (at any age) could lead to stigmatization and social rejection.

Brown and Mankowski (1993) also found that deficits in self-esteem can lead to issues with identity. Slomkowski et al. (1995) linked self-esteem and social functioning in those with ADHD to negative future outcomes. Luyckx et al. (2013) further contributed to the literature by finding a positive correlation between sense of identity and self-esteem.

Boer et al. (2019) conducted a longitudinal study in adolescents tracking their ADHD symptoms, social media use intensity, and problematic social media use and found that adolescents who had more problematic social media use had increased ADHD symptoms a year after initial collection. Dekkers and van Hoorn (2022) also found that adolescents who displayed symptoms of ADHD displayed more problematic and more frequent use of social media, further exploring the link between ADHD and social media. Social media use was also found to have a strong correlation with IP (Myers, 2021). Diaz (2022) notes in an article that dopamine is neurotransmitter more commonly linked to addiction and that social media has been found to trigger the dopamine reward system, leading to more frequent and problematic social media use.

The significance of this study was that multiple variables were brought together for the first time to explore the relationship between ADHD and identity distress as well as ADHD and IP. The findings are relevant to clinicians who are working with those with ADHD that also might be struggling with identity issues. Recognizing the role that self-esteem, masking, and social media use has on identity can help inform treatment and intervention plans.

## 5. Limitations and Future Research

There are certain limitations to this study that should be explicitly noted. This study was limited by its inability to directly obtain ADHD diagnoses and therefore was reliant on self-reporting measures of ADHD. The ASRS v1.1 is also meant to be a screen for ADHD rather than a diagnostic tool. Other factors that could cause similar symptoms of ADHD, such as depression, anxiety, post-traumatic stress disorder, or inadequate sleep, were also not assessed within this study.

The focus of ADHD symptom severity instead of diagnosis was a decision made due to a lack of resources to ensure prior diagnosis or to conduct testing and clinical interviews to be able to diagnose. The ASRS was chosen to determine ADHD symptom severity because it is a measure specifically for adults, and four questions were added in an attempt to be more compliant with DSM-5-TR criteria. This study found that based on their responses, 8.6% of the population would likely meet DSM-5-TR criteria for ADHD, which is close to the estimated expected percentage of the college student population, which has been calculated based on multiple studies and is about 2 to 8% (Green & Rabiner, 2012).

There was a 4.4% discrepancy between participants who reported having a formal ADHD diagnosis and those who scored high in ASRS ADHD symptom severity. Because there was no way to verify the formality of diagnoses, 8.6% of participants who endorsed ADHD symptoms were used in the analyses instead of the 13% of participants who reported receiving a formal diagnosis. Because of this limitation, the findings may not have been fully representative of the sample. Additionally, the scope of the study focused on symptom severity as opposed to formal ADHD diagnosis. Although, using symptom severity provided scalar data as opposed to categorical data, which allowed for more rigorous analyses. Therefore, ADHD diagnosis was not included in the formal analyses. However, it is important to consider the nuances of both symptom severity and formal diagnoses among participants and to potentially explore differences in symptom severity and symptom presentation. Future directions should aim to include both symptom severity and formal diagnosis in the analyses.

Future research should attempt to recruit participants with a diagnosis of ADHD from a reputable source, who are able to provide evidence of said diagnosis, or provide assessments and clinical interviews to determine if a participant meets diagnostic criteria for ADHD, especially because many other factors could impact inattentiveness or hyperactivity at a certain point in time, such as anxiety, depression, sleep deprivation, caffeine intake, and others. However, this study attempted to be more stringent in determining those who might meet diagnostic criteria by including additional questions that represented criteria from the DSM-5-TR.

This study was also limited to college students, which reduces the generalizability to those who are not in college. Data were collected through self-reporting measures, cross-sectionally, which limits the control of outside factors. Participants’ total social media use, such as how often and which platforms/how many platforms, was not measured, as well as the longevity of their social media use, nor their motivation behind their usage. All of those factors could be responsible for differences in self-esteem, identity distress, and IP. In addition, due to the correlational nature of this cross-sectional collection of data, causation cannot be assumed. While significant gender differences were found in preliminary analyses, an exploratory full model analysis showed no significant gender differences between men and women. Having a more even distribution of genders could possibly reveal significant findings in the overall model, but currently there is a larger female to male ratio of participants.

Finally, future research should also attempt to view the long-term effects of social media on ADHD symptoms, self-esteem, identity distress, and IP to determine if earlier social media use in life makes a difference in the outcome of these variables. This project examined current social media use and did not explore how long someone had been using social media. Future research should explore if the type of content viewed on social media has an impact on the results. A longitudinal design could better answer many of these questions.

## Figures and Tables

**Table 1:** Correlation Matrix of Age and All Study Variables.

	1	2	3	4	5	6
1. Age	-					
2. ADHD Symptom Severity	−0.05	-				
3. Imposter Phenomenon	−0.05	0.50 ***	-			
4. Self-Esteem	0.02	−0.37 ***	−0.65 ***	-		
5. Identity Distress	−0.10 *	0.39 ***	0.46 ***	−0.43 **	-	
6. Masking	−0.05	0.42 **	0.52 ***	−0.50 **	0.39 **	-
7. Social Media Use	−0.25 ***	0.23 ***	0.18 ***	−0.10 *	0.28 ***	0.16 ***

* *p* < 0.05, ** *p* < 0.01, *** *p* < 0.001.

**Table 2:** *t*-Test Differences Between Men and Women.

	Males		Females		*t*	*p*	Cohen’s *d*
	*M*	*SD*	*M*	*SD*			
ADHD Symptom Severity	7.65	4.91	8.59	4.76	−2.03	0.043	4.81
Imposter Phenomenon	1.91	0.69	2.09	0.78	−2.57	0.010	0.75
Self-Esteem	2.84	0.54	2.77	0.56	1.42	0.156	0.55
Identity Distress	2.18	0.63	2.44	0.75	−3.89	<0.001	0.71
Masking	2.38	0.39	2.46	0.75	−1.95	0.026	0.44
Social Media Use	2.78	0.71	3.23	0.69	−6.85	<0.001	0.69

**Table 3:** *t*-Test Differences Between Those Who Meet Criteria for ADHD and Those Who Do Not.

	Does Not Meet		Meets Criteria		*t*	*p*	Cohen’s *d*
	*M*	*SD*	*M*	*SD*			
Imposter Score	1.99	0.73	2.63	0.74	−5.47	<0.001	−0.87
Self-Esteem	2.81	0.55	2.40	0.55	4.75	<0.001	0.76
Identity Distress	2.32	0.69	2.87	0.47	−4.80	<0.001	−0.77
Total Masking	2.42	0.44	2.82	0.47	−5.65	<0.001	−0.90
Social Media	3.01	0.71	3.30	0.80	1.68	0.027	0.31

**Table 4:** Standardized Path for Model.

Path	Estimate	Standard Estimate	*p*
ADHD Symptom Severity → Self-Esteem	−0.01	0.01	<0.001
Self-Esteem → Masking	−0.41	0.03	<0.001
Masking à Social Media Emotional Connection	0.29	0.08	<0.001
Social Media Emotional Connection → Imposter Phenomenon	0.21	0.10	0.028
Social Media Emotional Connection → Identity Distress	0.36	0.08	<0.001
ADHD Symptom Severity → Imposter Phenomenon	0.35	0.03	<0.001
ADHD Symptom Severity → Identity Distress	0.18	0.02	<0.001

## Data Availability

Data are unavailable due to privacy restrictions.

## Abbreviations

The following abbreviations are used in this manuscript:

ADHD	Attention-Deficit/Hyperactivity Disorder
ANOVA	Analysis of Variance
APA	American Psychological Association
ASD	Autism Spectrum Disorder
DSM-5-TR	Diagnostic and Statistics Manual 5th Edition, Text Revised
CAT-Q	Camouflaging Autist Traits Questionnaire
CFI	Comparative Fit Index
CI	Confidence Interval
CMIN/DF	Chi-Squared Minimum/ Degrees of Freedom Ration
DSM-5-TR	Diagnostic and Statistics Manual of Mental Disorders, 5th Edition, Text Revision
IP	Imposter Phenomenon
IRB	Institutional Review Board
RMSEA	Root-Mean-Square Error of Approximation
SEM	Structural Equation Model
SNS	Social Networking Sites
TLI	Tucker–Lewis Index
UCF	University of Central Florida
